# Drug combination screening as a translational approach toward an improved drug therapy for chordoma

**DOI:** 10.1007/s13402-021-00632-x

**Published:** 2021-09-22

**Authors:** Susanne Scheipl, Michelle Barnard, Birgit Lohberger, Richard Zettl, Iva Brcic, Bernadette Liegl-Atzwanger, Beate Rinner, Claudia Meindl, Eleonore Fröhlich

**Affiliations:** 1 Department of Orthopaedics and Trauma, Medical University of Graz, Graz, Austria; 2 Cancer Research UK – AstraZeneca Antibody Alliance Laboratory, Cambridge, UK; 3 Diagnostic and Research Institute of Pathology, Medical University of Graz, Graz, Austria; 4 Division of Biomedical Research, Medical University of Graz, Graz, Austria; 5 Center for Medical Research, Medical University of Graz, Graz, Austria

**Keywords:** Bone tumour, Chordoma, Precision medicine, Targeted therapy, EGFR inhibitor, Combination screen

## Abstract

**Purpose:**

Drug screening programmes have revealed epidermal growth factor receptor inhibitors (EGFR_i_s) as promising therapeutics for chordoma, an orphan malignant bone tumour, in the absence of a known genetic driver. Concurrently, the irreversible EGFR_i_ afatinib (Giotrif®) is being evaluated in a multicentric Phase II trial. As tyrosine kinase inhibitor (TKI) monotherapies are invariably followed by resistance, we aimed to evaluate potential therapeutic combinations with EGFR_i_s.

**Methods:**

We screened 133 clinically approved anticancer drugs as single agents and in combination with two EGFR_i_s (afatinib and erlotinib) in the clival chordoma cell line UM-Chor1. Synergistic combinations were analysed in a 7 × 7 matrix format. The most promising combination was further explored in clival (UM-Chor1, MUG-CC1) and sacral (MUG-Chor1, U-CH1) chordoma cell lines. Secretomes were analysed for receptor tyrosine kinase ligands (EGF, TGF-α, FGF-2 and VEGF-A) upon drug treatment.

**Results:**

Drugs that were active as single agents (*n* = 45) included TKIs, HDAC and proteasome inhibitors, and cytostatic drugs. Six combinations were analysed in a matrix format: *n* = 4 resulted in a significantly increased cell killing (crizotinib, dabrafenib, panobinostat and doxorubicin), and *n* = 2 exhibited no or negligible effects (regorafenib, venetoclax). Clival chordoma cell lines were more responsive to combined EGFR-MET inhibition. EGFR-MET cross-talk (e.g. via TGF-α secretion) likely accounts for the synergistic effects of EGFR-MET inhibition.

**Conclusion:**

Our screen revealed promising combinations with EGFR_i_s, such as the ALK/MET-inhibitor crizotinib, the HDAC-inhibitor panobinostat or the topoisomerase-II-inhibitor doxorubicin, which are part of standard chemotherapy regimens for various bone and soft-tissue sarcomas.

**Supplementary Information:**

The online version contains supplementary material available at 10.1007/s13402-021-00632-x.

## Introduction

Chordomas are rare, primarily malignant bone tumours which typically arise in the axial skeleton, particularly in the clival and sacrococcygeal regions [1–3]. The tumours are slow-growing and usually present in an advanced stage of local disease [3]. The mainstay of treatment is surgery, as there is little benefit from conventional chemo- and radiotherapy [2]. Although particle therapy has emerged as an additional modality, particularly in the treatment of inoperable tumours and relapses [2], the prognosis for patients with chordoma is poor; the median survival is 7 years after diagnosis [1, 3]. If one considers irradiation damage of surrounding tissue and disabilities, which are often a consequence of surgical resections, then there is a strong case to develop new therapeutic options for this tumour entity [2, 3]. Currently, no targeted therapies, cytotoxic chemotherapies or immunotherapies are approved for chordoma [1, 2]. Cytotoxic chemotherapy has been demonstrated to be generally inactive in conventional chordomas, although isolated case reports have seen anecdotal responses in the ultra-rare subgroup of dedifferentiated chordomas [2, 4]. However, an increased in vivo effect of chemotherapeutic drugs in conventional chordomas has recently been reported if combined with the poly(ADP-ribose) polymerases (PARP)-inhibitor olaparib [5, 6]. Although some of the newly developed targeted therapies have revealed evidence of tumour response, overall such treatments appear to have limited benefit [2, 3, 7]: only moderate responses were observed in a non-randomised clinical trial with imatinib mesylate (Glivec®/Gleevec®, Novartis Pharma AG, Basel, Switzerland) [8] and in two non-randomised Phase II trials with anti-angiogenic multi-kinase inhibitors [2, 3, 7]. Consequently, several institutions have chosen to identify novel therapeutic targets empirically by testing diverse panels of compounds in a range of well-characterised chordoma models [7, 9–11]. The collective data from these phenotypic screens indicate that inhibitors of epidermal growth factor receptors (EGFRs) and erythroblastic leukaemia viral oncogene homologues (ErbBs) represent a group of compounds that are most effective against chordoma cell growth in vitro in the absence of common driver mutations in *EGFRs* and their downstream effectors [9–12]. In line with these findings, several isolated case reports have seen positive effects upon treatment with EGFR inhibitors [3, 7, 13]. Supported by these data, a European multicentre clinical trial involving a second-generation EGFR inhibitor (afatinib or Giotrif®, Boehringer Ingelheim, Ingelheim, Germany) is currently enrolling patients with advanced and metastasising chordoma (ClinicalTrials.gov Identifier: NCT03083678) [11]. Although the trial is still ongoing, it is well established that single-agent therapies have not yielded lasting effects, irrespective of cancer and treatment types: tyrosine kinase inhibitors (TKIs) face a diverse landscape of inter- and intra-tumoral heterogeneities; additionally, tumoral evolutions arising from selective pressures lead to intrinsic and acquired polyclonal resistance [14, 15]. Consequently, in a bench-to-bedside approach, the current translational study aims to identify targets that synergise with EGFR/ErbB inhibitors to overcome resistance issues and, thereby, increase and prolong treatment effects for patients with advanced chordoma. We undertook a combination screen in the UM-Chor1 clival chordoma cell line and tested a panel of 133 US Food and Drug Administration (FDA)-approved anticancer drugs in combination with two EGFR/ErbB inhibitors (afatinib and erlotinib). The most promising compound class was then further analysed in other well-established clival and sacral chordoma cell lines.

## Materials and methods

### Cell culture

We utilised only well-established chordoma cell lines: the clival lines UM-Chor1 and MUG-CC1 as well as the sacral lines MUG-Chor1 and U-CH1. The chordoma cells were cultured in Iscove/RPMI 4:1 (Life Technologies, Carlsbad, CA, USA) containing 10% foetal bovine serum (Invitrogen, Carlsbad, CA, USA), 1% insulin, transferrin and sodium selenite (Life Technologies), 2 mM glutamine and 1% penicillin/streptomycin (Life Technologies). The chordoma cells were grown at a pH of 7.4 until they reached 80% confluency and were detached from the flasks using TrpLE Express (Invitrogen). All cells were grown in a 5% CO_2_ atmosphere at 37 °C. They were periodically checked for mycoplasma infection by polymerase chain reaction and authenticated by short tandem repeat analysis using a PowerPlex 16 System kit (Promega, Madison, WI, USA; Suppl. Table 1).

### Ethics approval

Ethical approval was obtained from the research ethics committee of the Medical University of Graz (reference number 32–138 ex 19/20).

### Test compounds

We tested a panel of 133 FDA-approved anticancer drugs that was kindly provided to us by the National Institute of Health (NIH) Cancer Institute’s Developmental Therapeutics Programme (DTP), Bethesda, Maryland, USA (Suppl. Table 2). The drugs were screened in UM-Chor1, a clival chordoma cell line sensitive to EGFR_i_s [9]. The anticancer compounds were diluted from 10 mM stocks (100% DMSO) using a Versette pipetting robot (Thermo Fisher Scientific, Waltham, MA, USA) to create 1 mM compound plates (10% DMSO). All compounds were profiled alone and in combination with the irreversible EGFR_i_ afatinib (BIBW 2992; SelleckChem, Houston, TX, USA) or the reversible EGFR_i_ erlotinib (SelleckChem).

### Compound profiling and combination screen

**Fig. 1:**
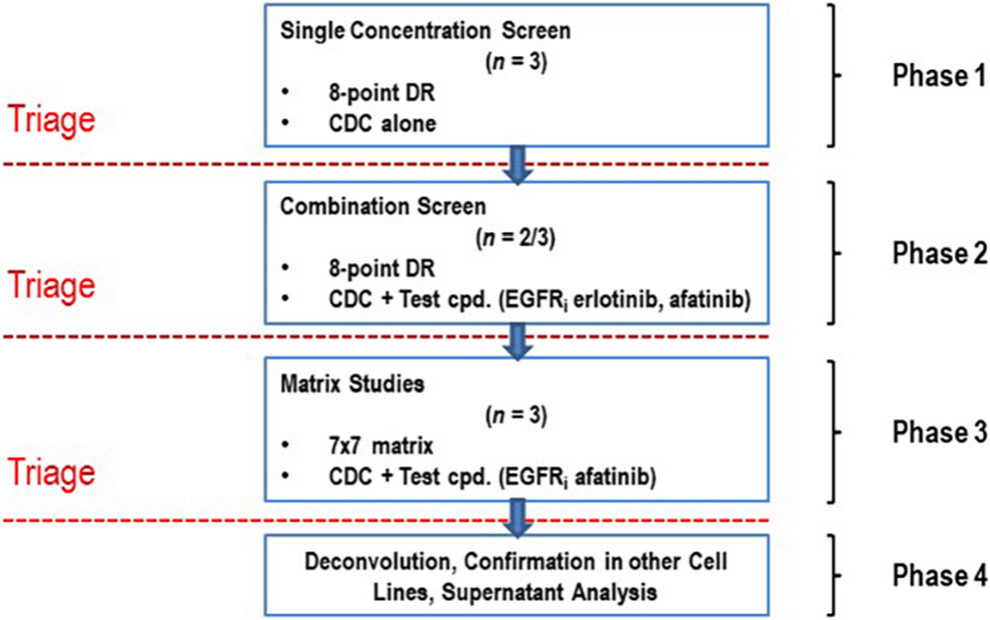
Flow diagram illustrating the screening procedure. DR: dose response. CDC: co-dosing compound = anticancer drug. Test cpd.: test compound = EGFR_i_ (erlotinib and afatinib). EGFR_i_: EGFR/ErbB inhibitor (erlotinib and afatinib)

First, we optimised and standardised our pre-test conditions for signal windows, numbers of cells seeded per well, edge effects, and toxicities of the solvent dimethyl sulfoxide (DMSO; Sigma Aldrich, St. Louis, MO, USA; data not shown). The half-maximal effective concentration (EC_50_) was determined for afatinib (0.16 μM) and erlotinib (0.4 μM) in an 8-point dose-response format. For the screen, we first tested the anticancer drugs alone in an 8-point dose-response format (20 μM to 0.3 nM) using a 96-well plate layout (nine compounds per plate; Fig. 1): 20x mother plates were prepared by conducting 1:5 serial dilutions for each test compound. Relevant controls, including staurosporine (SelleckChem), DMSO (Sigma) and media blanks were included on each plate (minimum *n* = 4 technical replicates). To create the final assay plates, 5 × 10^3^ cells were seeded in medium (90 μl/well) using a Multidrop Combi liquid dispenser (Thermo Fisher Scientific, Waltham, MA, USA) and cultured for 24 h before the compounds were added using a Versette pipetting robot (10 μl/well). To ensure reproducibility and comparability with the subsequent combination studies, the EC_50_s of afatinib and erlotinib were monitored on each assay plate (minimum of *n* = 4 technical replicates). After 96 h of incubation, CellTiter-Glo® Cell Viability Luminescent Assay (CTG; Promega, Walldorf, Germany and Madison, WI, USA) was added, and the plates were read in a LUMIstar Plate Reader (BMG Labtech, Aylesbury, UK). In the next step (Fig. 1), we conducted 8-point dose-response formats for each anticancer compound as described above. The EGFR_i_s afatinib or erlotinib were added in their EC_50_s (0.16 μM and 0.4 μM, respectively) simultaneously to each well. After 96 h, CTG was added, and the plates were read as outlined above. For each experiment, at least two biological replicates were conducted. If a combination appeared to be synergistic, then a third replicate was performed.

### Data analysis and hit selection

Percentages of inhibition and standard deviations were calculated from raw data relative to the controls on each plate. Growth curves were calculated using XLfit v. 5.0 (IDBS, Guildford, UK). Synergistic combinations were selected based on a minimum shift in potency of 20% upon addition of an EGFR_i_ (either afatinib or erlotinib; compound alone versus compound plus EGFR_i_).

### Matrix studies

Combinations that indicated synergistic trends were advanced into 7 × 7 matrix experiments to study synergistic dose profiles in more detail (Fig. 1). Matrix studies were conducted with the EGFR_i_ afatinib, as this TKI is currently being tested in a clinical multicentre trial and, thus, is more clinically relevant than erlotinib [11]. Both the DTP-anticancer compound and afatinib were studied alone and in combination with seven increasing concentrations (20 μM to 0.00128 μM = 1.28 nM) of their respective partner in a 96-well format. Compound concentrations were adapted for panobinostat, as this drug is highly potent, so that matrix studies were conducted in a lower concentration range (0.032 μM to 2.05 × 10^−6^ nM). Cells were seeded using a Multidrop Combi liquid dispenser, incubated for 24 h, and treated with the anticancer compound and afatinib using a Versette pipetting robot. After 96 h of incubation, CTG was added, and the plates were read using a LUMIstar Plate Reader according to the manufacturer’s recommendations. Statistical analysis was conducted using the student’s t test in GraphPad version 8.0 (GraphPad Software, San Diego, CA, USA). *P* values ≤ 0.05 (*) were considered significant.

### xMAP human bone metabolism magnetic bead panel

Using a Luminex® xMAP® platform in a magnetic bead format, we simultaneously studied the following analytes from the culture supernatants of MUG-Chor1 and UM-Chor1 cells: epidermal growth factor (EGF), tumour necrosis factor alpha (TNFα), vascular endothelial growth factor (VEGF), and fibroblast growth factor 2 (FGF2). No cross-reactivity was noted between the antibodies for an analyte or between any of the other analytes in this panel. Treatment was performed with 4 μM crizotinib (PF-02341066; SelleckChem, Houston, TX, USA), 0.16 μM afatinib (BIBW2992; SelleckChem), or with a simultaneous co-dosing of crizotinib and afatinib for 72 h. For detection, we used a commercially available Procarta Plex (Thermo Fisher, Waltham, MA, USA) on a Bioplex200 system (Bio-Rad Laboratories, Hercules, CA, USA) according to the manufacturers´ instructions. Measurement of mean fluorescence intensities was performed using Bio-Plex Manager software, version 4.1 (Bio-Rad Laboratories, Hercules, CA, USA) with a 5-parametric curve fitting.

## Results

### Single-agent screening of 133 FDA-approved anticancer drugs in UM-Chor1 cells confirms activity of specific compound classes

First, we examined the dose-response profiles of 133 FDA-approved anticancer compounds if these had been tested as single agents. Most compounds were classified as inactive (*n* = 67; Suppl. Table 3). Amongst these drugs were various cytostatic drugs (*n* = 33), hormone blockers (*n* = 8), immunomodulatory antineoplastic agents (*n* = 3: thalidomide, lenalidomide and pomalidomide) and photoactive drugs (*n* = 2). This group also comprised all PARP inhibitors included in the drug set (*n* = 3: olaparib, rucaparib and niraparib) as well as a certain proportion of TKIs (*n* = 11). The latter particularly included VEGF receptor (VEGFR) and multi-kinase inhibitors (*n* = 4: lenvatinib, axitinib, sunitinib and pazopanib) as well as BCR-ABL inhibitors (*n* = 2: ponatinib and nilotinib), hepatocyte growth factor receptor (HGFR, c-MET and MET) inhibitors (*n* = 2: crizotinib and cabozantinib) as well as inhibitors of hedgehog signalling (vismodegib), the BRAF proto-oncogene(vemurafenib) and the chemokine receptor CXCR4 (plerixafor). Inactive drugs included *n* = 7 drugs of various other modes of action, such as the non-steroidal, anti-inflammatory drug celecoxib, the immune response modifier imiquimod, the cytoprotectant amifostine, the differentiating agent tretinoin, the iron chelator dexrazoxane, the uricostatic agent allopurinol and the bisphosphonate zoledronic acid.

A fair proportion of compounds exerted partial activity at high compound concentrations (> 1 μM; *n* = 21; Suppl. Table 3). In addition to various cytostatic drugs (*n* = 7), this class contained several TKIs (*n* = 12) as well as the selective oestrogen receptor modulator tamoxifen and the synthetic oestrogen receptor antagonist fulvestrant. Amongst the TKIs in this category were the multi-kinase inhibitors imatinib, sorafenib and regorafenib. Other TKIs exerting partial activity at high concentration ranges included inhibitors of various types of kinases: the anaplastic lymphoma kinase ALK (alectinib), the small molecule smoothened/hedgehog (erismodegib), phosphoinositide-3 kinase (idelalisib), Src (dasatinib), EGFR/ErbB (gefitinib), BRAF (dabrafenib) or B cell lymphoma 2 (Bcl-2; venetoclax). Furthermore, this group included the cyclin-dependent kinase 4/6 inhibitors ribocliclib and palbociclib.

**Fig. 2:**
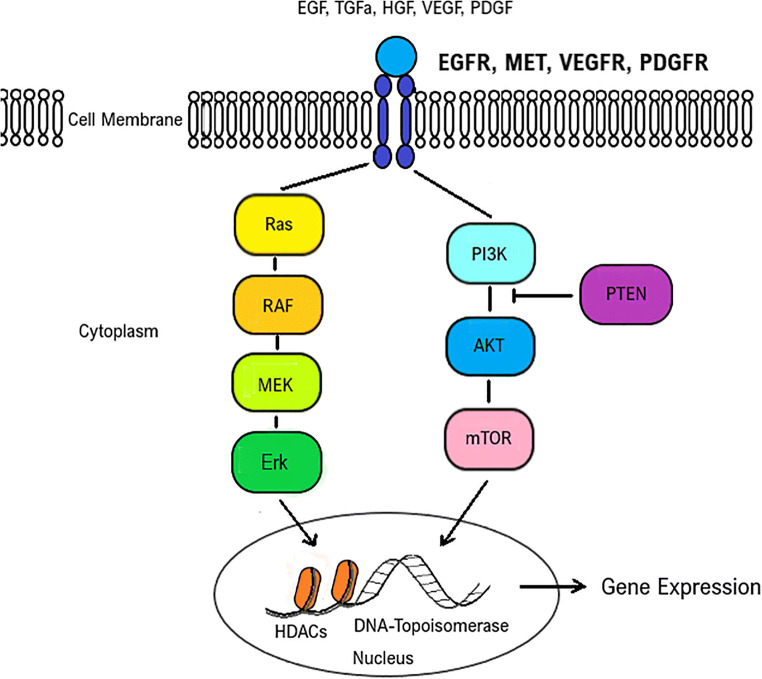
Illustration of drug targets identified in the screen. Upon activation by growth factors, transmembrane receptor tyrosine kinases (RTKs) utilise common intracellular signalling pathways to modulate intracellular activities and gene expression levels. Examples of RTKs include the epidermal growth factor (EGF) receptor (EGFR), the hepatocyte growth factor (HGF) receptor (HGFR, MET), the vascular endothelial growth factor (VEGF) receptor (VEGFR) and the platelet-derived growth factor (PDGF) receptor (PDGFR) [16–20]. Common intracellular RTK downstream signalling pathways are, e.g., the rat sarcoma (Ras)- rapidly accelerated fibrosarcoma (RAF)- mitogen-activated protein kinase (MEK)- extracellular signal-regulated kinase (Erk) pathway, and the phosphatidylinositol-3-kinase(PI3K)– protein kinase B (Akt)– mammalian target of rapamycin (mTOR) pathway [14, 15, 21]. Phosphatase and tensin homolog (PTEN), a tumour suppressor, negatively regulates Akt/mTOR signalling [19, 22]. Histone deacetylases (HDACs) modify DNA-packaging and alter its accessibility for transcription factors [23, 24]. DNA topoisomerases modify the tertiary DNA conformation and are involved in DNA replication [25]

A total of *n* = 45 compounds was classified as active (Suppl. Table 3). These included cytostatic drugs (*n* = 25), histone deacetylase (HDAC) inhibitors (*n* = 4: vorinostat, panobinostat, belinostat and romidepsin) (Fig. 2), proteasome inhibitors (*n* = 3: bortezomib, ixazomib citrate and carfilzomib) and TKIs (*n* = 13). Active cytostatic drugs included, for example, doxorubicin (Fig. 2), idarubicin, vinorelbine, vincristine, vinblastine and etoposide. Amongst TKIs exerting activity in UM-Chor1, the majority were EGFR/ErbB inhibitors (*n* = 5: afatinib, erlotinib, osimertinib, vandetanib and lapatinib) (Fig. 2). Other active TKIs were inhibitors of the mammalian targets of rapamycin (mTOR; *n* = 3: sirolimus, everolimus and temsirolimus), all displaying cytostatic activity, and the mitogen-activated protein kinase kinase 1 (*n* = 2: cobimetinib and trametinib) (Fig. 2). Furthermore, activity was observed for ibrutinib, an inhibitor of Bruton’s tyrosine kinase, the dual ABL- and SRC-inhibitor bosutinib, and the ALK-inhibitor ceritinib.

### EGFR/ErbB inhibitors increase the potency of FDA-approved anticancer drugs

We next combined the EGFR_i_s afatinib and erlotinib with each of these 133 anticancer agents. The EC_50_ values for the EGFR_i_s used in the subsequent combination studies of afatinib and erlotinib were 0.16 μM and 0.4 μM, respectively. This is comparable to our previous experiments and confirms a satisfactory response to EGFR_i_s in UM-Chor1 cells [9]. As reported previously, afatinib showed a biphasic curve profile [9].

**Fig. 3:**
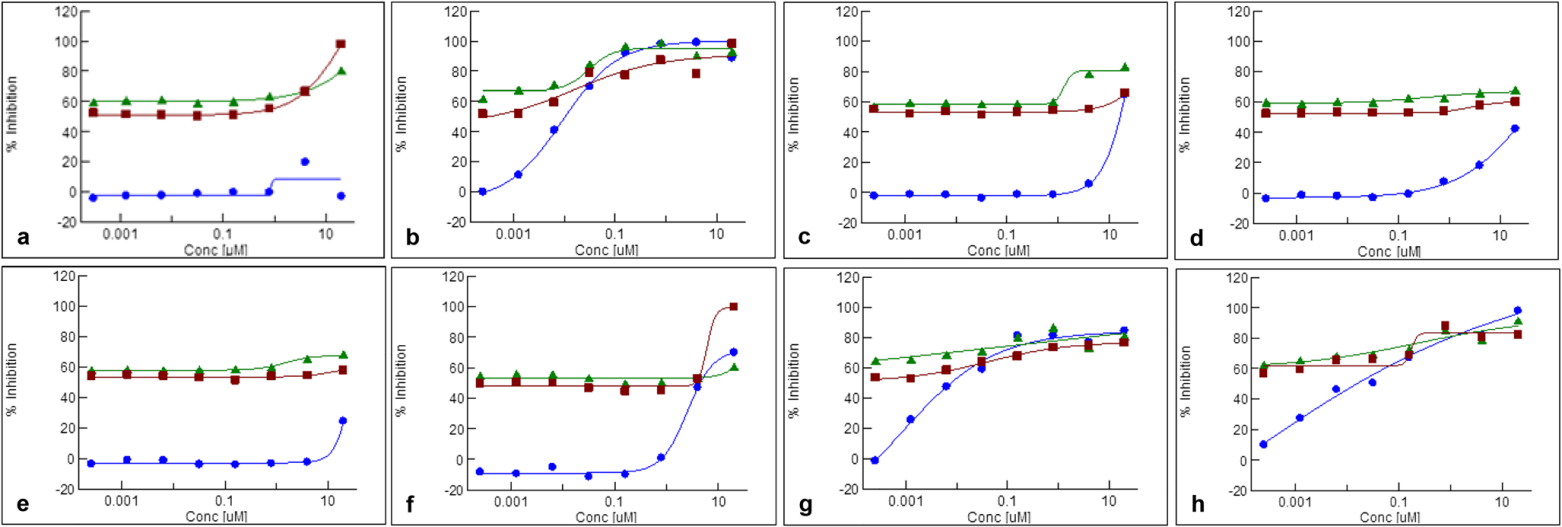
Composite of screening profiles of *n* = 8 anticancer drugs that displayed an increased cytotoxicity (minimum plus 20% cell killing at any test concentration) upon combination with an EGFR_i_ (erlotinib and afatinib) in the clival chordoma cell line UM-Chor1: crizotinib (**a**), panobinostat (**b**), venetoclax (**c**), dabrafenib mesylate (**d**), regorafenib (**e**), bosutinib (**f**), doxorubicin hydrochloride (**g**) and idarubicin hydrochloride (**h**). Blue: anticancer drug = co-dosing compound (CDC) as a single agent. Green: CDC in combination with the EGFR_i_ afatinib (simultaneous dosing). Red: CDC in combination with the EGFR_i_ erlotinib (simultaneous dosing)

Compared to their potencies as single agents, we observed an increase in potency (plus 20%) in *n* = 8 anticancer drugs if they were combined with an EGFR_i_(Table 1; Fig. 3). To ensure that we did not miss a potential hit in this step, we considered drugs that exerted differences in potencies at any concentration range, irrespective of its spectrum and width. Shifts in potency were observed for TKIs (*n* = 5: crizotinib, venetoclax, dabrafenib, regorafenib and bosutinib), one HDAC inhibitor (panobinostat), and two cytostatic drugs (doxorubicin hydrochloride and idarubicin hydrochloride, both DNA-topoisomerase inhibitors; Fig. 3). Similar results were obtained for combinations with afatinib and erlotinib (Fig. 3 and Suppl. Table 3).

**Table 1:** Anticancer drugs (*n* = 8) exerting a shift in potency in at least one test concentration (minimum plus 20%) upon combination with an EGFR_i_. ALK: anaplastic lymphoma kinase. MET: MET proto-oncogene, c-MET. ROS1: ROS proto-oncogene 1. HDAC: histone deacetylase. BRAF: B-Raf proto-oncogene. Bcl-2: B cell lymphoma 2. SRC: SRC proto-oncogene. ABL: ABL proto-oncogene 1

Compound Name	Target	Drug Name*
		(*examples)
Crizotinib	ALK, MET and ROS1	XALKORI, Pfizer Inc., New York City, NY, USA
Panobinostat	HDAC isoforms I, II and IV	FARYDAK, Novartis, Basel, Switzerland
Doxorubicin hydrochloride	DNA-topoisomerase II	ADRIAMYCIN, Pfizer Inc.; Caelyx; Myocet; and others
Dabrafenib mesylate	(Mutated) BRAF kinases	TAFINLAR, GlaxoSmithKline, London, UK
Venetoclax	Bcl-2	VENCLEXTA (USA), AbbVie Inc., Lake Bluff, IL, USA and Roche Genentech Inc., South San Francisco, CA, USA
Regorafenib	Multi-kinase inhibitor	STIVARGA, Bayer HealthCare Pharmaceuticals Inc., Leverkusen, Germany
Bosutinib	SRC/ABL tyrosine kinase	BOSULIF, Pfizer Inc.
Idarubicin hydrochloride	DNA-topoisomerase II	IDAMYCIN (USA), Pfizer Inc.

### The MET/ALK-inhibitor crizotinib induces a significantly increased cytotoxicity in combination with the EGFR-inhibitor afatinib

**Fig. 4:**
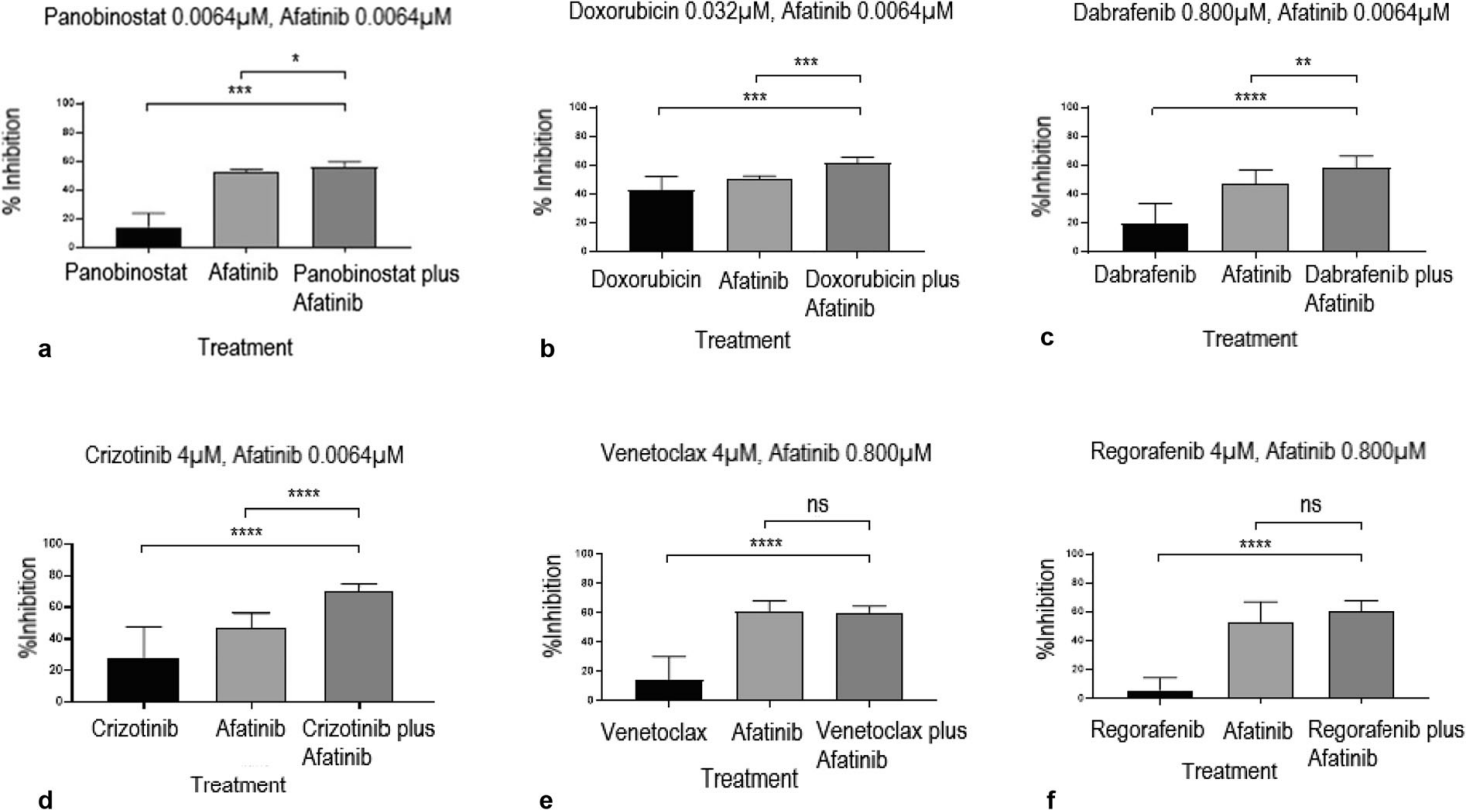
Statistical analysis of matrix screening results of *n* = 6 anticancer drugs (panobinostat, doxorubicin, dabrafenib, crizotinib, regorafenib and venetoclax) in combination with the EGFR_i_ afatinib. *P* values ≤ 0.05 are considered significant (*p = 0.01 to 0.05; **p = 0.001 to 0.01; ***p = 0.0001 to 0.001; ****p < 0.0001; ns: *p* ≥ 0.05; non-significant). Bar graphs illustrate percentages of inhibition (defined as reduction in cell viability) obtained with the anticancer drugs panobinostat (0.0064 μM; **a**), doxorubicin (0.032 μM; **b**), dabrafenib (0.800 μM; **c**) and crizotinib (4 μM; **d**), and the EGFR_i_ afatinib (0.0064 μM), alone and in combination. Venetoclax (**e**) and regorafenib (**f**) do not induce significantly increased cell killing at comparable concentrations (anticancer drug ≤ 4 μM; afatinib ≤ 1 μM)

In the next step, we investigated dosing profiles and dose ranges of synergistic combinations. Therefore, six drugs were advanced into 7 × 7 matrix studies: crizotinib, panobinostat, venetoclax, dabrafenib mesylate, regorafenib and doxorubicin (Fig. 4). Panobinostat as well as doxorubicin showed a high potency in UM-Chor1 cells (Suppl. Table 3). Both drugs exerted synergy at comparatively low concentrations (Fig. 4a and b): panobinostat yielded moderate synergy at dose ranges of 0.0064 μM or below (Fig. 4a). Similarly, doxorubicin exerted synergy at 0.032 μM or below (Fig. 4b). The other drugs (crizotinib, venetoclax, dabrafenib and regorafenib) presented low potencies as single agents (Suppl. Table 3). We observed synergistic trends for dabrafenib at a medium dose range (4 μM to 0.160 μM) in combination with low doses of afatinib (0.032 μM or below; Fig. 4c). For regorafenib, a similar trend was observed, although it was less pronounced and non-significant(Fig. 4f). Venetoclax revealed a minor but non-significant trend at its top concentrations (20 μM and 4 μM, respectively; Fig. 4e). Crizotinib (4 μM) yielded significantly increased cytotoxic effects in combination with afatinib (0.8 μM or lower; *p* < 0.0001; Fig. 4d).

### The combination of crizotinib and afatinib is superior to single-agent therapy in clival chordoma cells

**Fig. 5:**
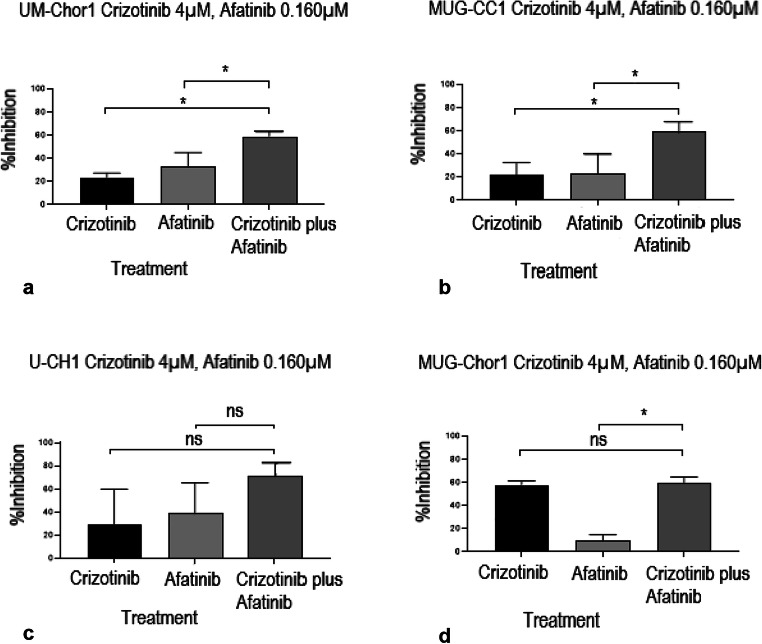
Combination of the ALK/MET-inhibitor crizotinib (4 μM) and the EGFR_i_ afatinib (0.160 μM) significantly increases cell killing compared to the respective single agents in clival chordoma cell lines (UM-Chor1 and MUG-CC1;**a** and **b**), whereas this effect is less distinct in sacral chordoma cell lines (U-CH1 and MUG-Chor1;**c** and **d**). *p < 0.05; ns: *p* ≥ 0.05; non-significant

The combination of afatinib and crizotinib exerted the most distinct cytotoxic effect on UM-Chor1 cells, Consequently, we next investigated whether a similar effect could be seen in other chordoma cell lines. We therefore conducted 7 × 7 matrix studies for crizotinib and afatinib in four chordoma cell lines: two clival (UM-Chor1 and MUG-CC1) and two sacral (U-CH1 and MUG-Chor1). We found that afatinib was active in all cell lines as a single agent (Suppl. Fig. 1). Crizotinib demonstrated low activity in all four cell lines as a single agent: the best response for this drug was observed in the sacral cell line MUG-Chor1(Suppl. Fig. 1). As illustrated in Fig. 5, we observed a significant increase in cytotoxicity with the combination of crizotinib and afatinib in the clival cell lines UM-Chor1 and MUG-CC1, but not in the sacral cell lines U-CH1 and MUG-Chor1(Fig. 5).

### Autocrine TGF-α and VEGF-A secretion indicate TGF-α/EGFR and VEGF-A/VEGFR cross-talk with HGF/c-MET signalling

**Fig. 6:**
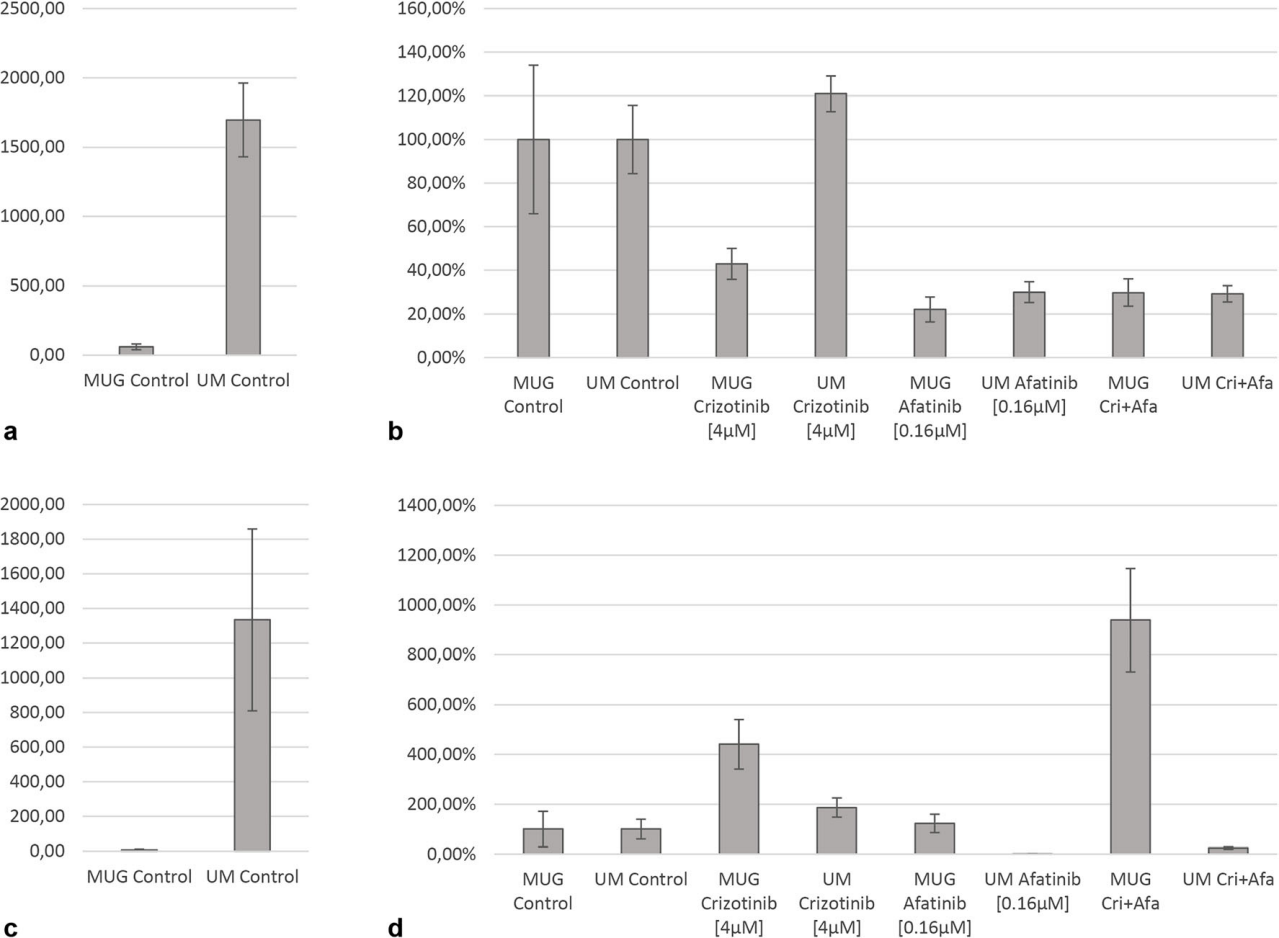
Detection of the receptor tyrosine kinase ligands TGF-α and VEGF-A in supernatants of the clival chordoma cell line UM-Chor1 and the sacral chordoma cell line MUG-Chor1 using a Luminex® xMAP® platform. Figures **a** and **c** display bar charts of the expression via absolute fluorescence intensity measurements of TGF-α (**a**) and VEGF-A (**c**) in the untreated control groups of both cell lines (UM-Chor1 and MUG-Chor1): UM-Chor1 cells show a higher baseline secretion of TGF-α, which is a ligand activating the EGF-receptor (**a**), and VEGF-A, which is a ligand activating the VEGF-receptor 2 (KDR; **c**) compared to MUG-Chor1 cells. Figures **b** and **d** present bar charts of relative fluorescence intensity measurements after normalisation in the untreated control group of each cell line (UM-Chor1 and MUG-Chor1). Upon treatment with the ALK/MET-inhibitor crizotinib, UM-Chor1 cells markedly increase their TGF-α secretion (**b**). This effect is not observed in MUG-Chor1 cells and suggests that EGFR signalling is activated to compensate for the ALK/MET blockade in UM-Chor1 cells (**b**). In line with this, TGF-α secretion is persistently low upon combined treatment with the EGFR_i_s afatinib and crizotinib (**b**). Conversely, MUG-Chor1 cells exhibit a marked increase in VEGF-A secretion (**d**). Increased secretion of VEGF-A was seen upon treatment with both crizotinib and afatinib separately, but particularly with their combination (**d**). This effect indicates that VEGFR signalling is activated to compensate for EGFR and ALK/MET blockade in MUG-Chor1 cells

We next asked whether there was evidence for autocrine activation of the EGFR pathway in UM-Chor1, a clival cell line responsive to the crizotinib-afatinib combination, and MUG-Chor1, a sacral cell line irresponsive to this drug combination. To answer this question, we measured the secretion of two growth factors known to activate the EGF-receptor, i.e., EGF and transforming growth factor alpha (TGF-α), in the cells´ supernatants [16]. Additionally, we measured the secretions mean fluorescence intensities of fibroblast growth factor-2(FGF2) and vascular endothelial-derived growth factor A (VEGF-A), which activate the FGF-receptor 2 (FGFR2) and the VEGF-receptor 2 (VEGFR2 or KDR), respectively. Furthermore, VEGF-A and FGF2 are known to be involved in EGFR resistance and tumorigenesis [17, 18, 26]. All of these factors have previously been implicated in chordoma pathogenesis [27–29]. EGF and FGF2 were only detectable at extremely low levels in both cell lines (data not shown). In contrast, distinct signals could be obtained for TGF-α and VEGF-A: compared to MUG-Chor1, UM-Chor1 cells showed a high baseline secretion of TGF-α and VEGF-A (Fig. 6a and c). TGF-α secretion in UM-Chor1 cells was further increased upon treatment with crizotinib (Fig. 6b). Afatinib suppressed TGF-α secretion in both cell lines. TGF-α secretion was persistently low in both lines after combined treatment with afatinib and crizotinib. For VEGF-A, MUG-Chor1 cells showed a marked increase in secretion upon treatment with crizotinib and afatinib, especially with the combination of crizotinib and afatinib (Fig. 6d). In contrast, UM-Chor1 cells only showed a minor increase of VEGF-A secretion upon treatment with crizotinib, which was reversed when this drug was combined with afatinib (Fig. 6d).

## Discussion

Currently, no targeted therapies, cytotoxic chemotherapies or immunotherapies are approved for chordoma [1, 2]. In search of additional treatment options for patients with advanced stages of this disease, some TKIs have entered early-phase clinical trials or are prescribed to patients in compassionate usage [2, 7, 13]. However, single-agent therapies have not shown lasting effects, irrespective of cancer or treatment type. The benefits of these treatments only last for an average of 6 to 12 months, and resistance usually already occurs after 2 or 3 months [14, 15].

In our translational approach, we aimed to identify novel therapeutic agents for chordoma combination therapy to increase and prolong treatment effects of EGFR_i_s that are currently being administered to patients in clinical trials and in palliative, off-label usage [7, 11, 13]. To this end, we undertook a combination screen employing a panel of 133 FDA-approved anticancer drugs in combination with two EGFR_i_s to explore which drugs exerted synergistic effects upon chordoma cell killing in vitro.

We conducted this screen in UM-Chor1, which is a clival chordoma cell line that has been proven to be sensitive to EGFR_i_s [9–11]. The analysis of whole genome sequencing data revealed that this cell line carries a *PTEN* L139* nonsense mutation [12], which has also been described in conventional glioblastoma, endometrial and breast cancers [30]. Tarpey et al. did not detect any recurrent genetic drivers in their genetic analyses of 104 sporadic chordomas, but alterations in phosphoinositide-3 kinase signalling (including occasional mutations in *PIK3CA* and *PTEN*) were amongst the more common genetic events reported [12]. Furthermore, several authors have previously described heterozygous losses of *PTEN* in chordomas [19, 22, 31], whereas others have implicated a role of mTOR and MAPK signalling in chordoma pathogenesis [21]. Subsequently, non-randomised phase II chordoma trials explored a combined treatment with imatinib and a mTOR inhibitor, but yielded moderate successes [7, 32]. Given the lack of currently druggable targets, phenotypic testing of drugs and their combinations may yield novel therapeutic strategies and address heterogeneities of diseases that have not been sufficiently covered by target-based approaches to date [33].

We first tested a panel of 133 FDA-approved anticancer drugs as single agents. All of these drugs were then re-tested in combination with two small molecule EGFR_i_s: the irreversible inhibitor afatinib (Giotrif®), as it is currently being evaluated in a European multicentric Phase II study, and the reversible inhibitor erlotinib (Tarceva®, Roche, Basel, Switzerland), as it has presented promising effects in several well-documented case reports [7, 13]. As expected, most compounds were inactive as single agents (*n* = 67) or exerted only moderate activities at high (and thus, likely toxic) concentrations (*n* = 21). We found that all PARP inhibitors included in this drug set (olaparib, rucaparib and niraparib) were amongst these inactive drugs. Groschel et al. [5] have described a signature of defective homologous recombination DNA repair in advanced chordomas. As PARP inhibitors are particularly toxic to DNA-repair incompetent cells, Groschel et al. hypothesised that PARP inhibitors may be potential targets for chordoma treatment [5]. These inhibitors proved inactive as single agents in the current study and in our previous screen [9]. However, PARP inhibitors are likely to exert their effects particularly in combination with other chemotherapeutic drugs, as has previously been suggested by several authors [6, 34]. This aspect, which still requires further investigation, once again underscores the translational importance of well-conducted, extensive combination screens for this orphan disease.

The single-agent screen yielded *n* = 45 compounds which were classified as active. Most of them were TKIs inhibiting EGFR/ErbBs or downstream effectors of various receptor tyrosine kinases, such as mTOR or MAPK. This sensitivity to EGFR_i_s, and particularly the downstream effectors mTOR and MAPK, may be influenced by the *PTEN* L139* mutation seen in this cell line. Apart from TKIs, HDAC (e.g. panobinostat) and proteasome (e.g. bortezomib) inhibitors were also highly active in UM-Chor1 cells. Previously, several research groups have taken an interest in HDACs and their inhibition in chordoma models [19, 23, 35]. In an extensive compound screen, however, neither HDAC nor proteasome inhibitors showed chordoma-selective cytotoxicities. Consequently, these drug classes were not addressed again [9]. The same applies for several chemotherapeutic drugs exerting activities as single agents, including the topoisomerase-II-inhibitors doxorubicin, idarubicin and etoposide, as well as mitosis inhibitors such as vincristine and vinblastine [9].

Based on the selected cut-offs and the compounds’ curve profiles, we identified eight drugs that showed synergistic trends in combination with an EGFR_i_. These data agree with findings from our previous focussed compound screen, in which the targets of these drugs, including BRAF, SRC and VEGFR, were revealed to be co-targets of several hit compounds [9]. Subsequently, we tested six drugs in 7 × 7 matrix studies in combination with the EGFR_i_ afatinib: the multi-kinase-inhibitor regorafenib, the Bcl-2-inhibitor venetoclax, the HDAC-inhibitor panobinostat, the BRAF-inhibitor dabrafenib mesylate, the topoisomerase-II-inhibitor doxorubicin and the ALK/MET-inhibitor crizotinib. When profiled in a matrix format, four drugs exhibited a significantly increased cytotoxicity in combination with afatinib compared to their effects as single agents: panobinostat, doxorubicin, dabrafenib and crizotinib. However, venetoclax and regorafenib did not exert significant effects in a comparable, sub-maximal dose range.

The observation that the multi-kinase-inhibitor regorafenib failed to reveal significant synergistic effects in our matrix study agrees with previous reports of the limited activity of multi-kinase inhibitors in chordoma models [9] and their moderate successes in Phase II chordoma trials [7].

Given the *PTEN* mutation seen in this cell line, it is not surprising that the BRAF-inhibitor dabrafenib acted synergistically with EGFR/ErbB inhibitors in our combination screen and the subsequent matrix experiments.

Of note is that the HDAC-inhibitor panobinostat also exerted moderate synergy in combination with the EGFR_i_ afatinib. Previous studies have reported combined HDAC and platelet-derived growth factor receptor (PDGFR) inhibition to overcome *PTEN* disruption in chordoma [19]. However, to the best of our knowledge, combined EGFR and HDAC inhibition has not yet been studied in this orphan disease. HDAC inhibitors have not shown ground-breaking successes as mono-therapeutics in solid tumours including chordomas [36], but there seems to be a role for this class in combination therapy and multitarget inhibition [24, 36].

We furthermore observed synergies between EGFR_i_s and the chemotherapeutic drug doxorubicin, which is part of standard treatment regimens for various bone and soft-tissue sarcomas [25]. Although chordomas are chemo-resistant, there is anecdotal evidence of some activity in dedifferentiated and paediatric tumours [2]. Moreover, EGFR_i_s have been found to sensitise tumours, such as lung cancers, to chemotherapeutic agents [37], and combinations of EGFR_i_s and chemotherapeutic agents are being investigated in mesenchymal neoplasms, such as osteosarcomas [38]. We therefore hypothesise that a subset of chordoma patients may benefit from a combined EGFR_i_ and chemotherapeutic treatment.

Synergies observed with the ALK/MET-inhibitor crizotinib agree with existing data on both the target and the drug in chordoma and related diseases: several authors have reported expression of MET in a high proportion of clinical chordoma samples [39, 40] in the absence of recurrent MET mutations or amplifications [12]. Furthermore, MET signalling has been shown to act as a bypass signalling pathway and, thus, to confer resistance to EGFR_i_s [14, 20]. Previously, we reported this mechanism of resistance in another well-characterised sacral chordoma cell line, U-CH2 [9]. In line with our previous work [9], our current data indicate that crizotinib as a single agent is not potent in chordoma cells. However, the combination of crizotinib with the EGFR_i_ afatinib results in synergistic effects not only in chordoma cells resistant to EGFR inhibition, as reported previously for U-CH2 [9], but also in chordoma cells that respond to EGFR_i_s. These synergies are more pronounced in the clival (UM-Chor1 and MUG-CC1) than in the sacral (MUG-Chor1 and U-CH1) cell lines investigated here.

A possible explanation for this observation could be the increased secretion of TGF-α, which was seen in the clival cell line UM-Chor1 upon treatment with crizotinib. TGF-α is a known EGFR ligand and activator of EGFR signalling [16]. This effect, which was not observed in the sacral cell line MUG-Chor1, indicates a possible cross-talk of TGF-α/EGFR and HGF/c-MET signalling in UM-Chor1 cells. A similar EGFR/c-METcross-talk has previously been reported in other cancers, such as lung cancer [20]. In support of this hypothesis, combined treatment with afatinib and crizotinib markedly suppressed TGF-α secretion in our study and resulted in significantly increased UM-Chor1 cell killing. Furthermore, MUG-Chor1 cells showed a marked increase in VEGF-A secretion upon combined treatment with crizotinib and afatinib. It is therefore tempting to speculate that this increase of VEGF-A, being a key activator of VEGFR2, indicates the presence of cross-talk between VEGF-A/VEGFR and HGF/c-MET signalling in the sacral cell line MUG-Chor1. In particular, VEGFR/METcross-talk has been reported in other types of cancer and is thought to induce tumour neovascularisation [20, 26]. In this context, Bosotti et al. previously showed a high expression level of VEGFR2 (KDR) in MUG-Chor1 cells [41]. Tarpey et al. analysed whole-genome sequencing data of MUG-Chor1 cells and did not identify driver mutations in either *EGFR*, *MET*, *KDR* or other RTKs and related genes. The authors reported a duplication of *TBXT*, a homozygous deletion of *CDKN2A*, and a deletion of *TP53* [12]. These findings do not provide a molecular basis to suggest synergistic effects of EGFR/MET/VEGFR inhibition. Nevertheless, EGFRi exerted activity in the absence of *EGFR* mutations or mutated downstream effectors in MUG-Chor1 cells in a focused compound screen [9]. In the same screen, VEGFR1/2 signalling was identified as the pathway covering most of the non-EGFR target genes in a cell line panel including MUG-Chor1 [9]. However, comprehensive screening data on the activity of VEGFRi, METi, and compound combinations in MUG-Chor1 cells are lacking to date. Therefore, more in-depth work is required to identify potential synergistic combinations for this cell line, which is beyond the scope of our current study.

Given that single agent tyrosine kinase inhibitor therapies are not expected to show lasting effects [14], and facing heterogeneous drug responses, resistance mechanisms and compensatory strategies as reported by us and others [9, 11, 42], it seems reasonable to consider designing personalised (combination) treatment regimens for chordoma patients. The first evidence from well-documented case reports and clinical observations already indicates that chordoma patients may benefit from drug combinations [7, 43, 44]. Drug selection for (combination) treatment could, for instance, be based on comprehensive integrated approaches, which combine drug screens on patient-derived tumour cells, biomarker searches based on gene and gene expression analyses, or even multi-omics analyses of the patient’s (tumour) tissue, and in vivo studies [42, 45]. Currently, efforts are being made to develop such comprehensive integrated approaches for chordoma patients (personal communication).

A major limitation of this study is that we conducted this work on a limited number of cell lines, model systems, and drug combinations. Nevertheless, we have outlined a possible methodology to conduct drug combination screening in chordoma cell lines, which a wider research community can apply to a larger panel of disease models and compounds. Additionally, we have identified several promising synergistic combinations that fit with the existing literature and results from previous drug screens. We are convinced that combination screening as a translational approach will pave the way for improved personalised drug therapies, which are urgently sought for orphan diseases like chordoma.

## Supplementary Information

Suppl. Figure 1 The EGFR_i_ afatinib indicates activity in all four chordoma cell lines utilised in this study (UM-Chor1, MUG-Chor1, U-CH1, and MUG-CC1). EC_50_ values vary between cell lines but are all within the nanomolar or low micromolar range. The ALK/MET-inhibitor crizotinib is less potent as a single agent than afatinib. The EC_50_s of crizotinib are within a high-micromolar range in all four cell lines. (PNG 487 kb)

High resolution image (TIF 674 kb)

Suppl. Table 1 STR authentication profiles of chordoma cell lines utilised in this study. (DOCX 15 kb)

Suppl. Table 2 Panel of 133 U.S. Food and Drug Administration (FDA)-approved anticancer drugs that was kindly provided to us by the National Institute of Health (NIH) Cancer Institute, Developmental Therapeutics Programme (DTP), Bethesda, Maryland, USA. NSC: NSC number (Cancer Chemotherapy National Service Centre number); identifying number assigned by DTP. CAS: CAS Registry Number. MW: molecular weight. (DOCX 40 kb)

Suppl. Table 3 Summary of 133 FDA-approved anticancer drugs screened in UM-Chor1. The list provides drug names and compound classes as well as screening data of these drugs as single agents and in combination with the EGFR_i_s erlotinib and afatinib. Cpd: compound. Nr: number. NSC: NSC number (Cancer Chemotherapy National Service Center number); identifying number assigned by DTP. CAS: CAS Registry Number. MW: molecular weight. MoA: mechanism of action. PubChem: https://pubchem.ncbi.nlm.nih.gov. EC_50_: half-maximal effective compound concentration. R (1, 2, 3, and 4): number of biological repeats of the experiment. μM: micromolar concentration range. nM: nanomolar concentration range. %I: percentage of inhibition as defined by cell viability as determined by the CellTiter Glo assay. Conc: concentration. Combi: combination. (XLSX 1786 kb)

## Data Availability

Details on the drug panel are provided in Suppl. Table 2. All screening data are provided in Suppl. Table 3.
