# Supplementary figures and images for: Drug combination screening as a translational approach toward an improved drug therapy for chordoma
Source: Cell Oncol (Dordr). 2021 Sep 22;44(6):1231–42. doi: 10.1007/s13402-021-00632-x (PMC8648636; doi:10.1007/s13402-021-00632-x)

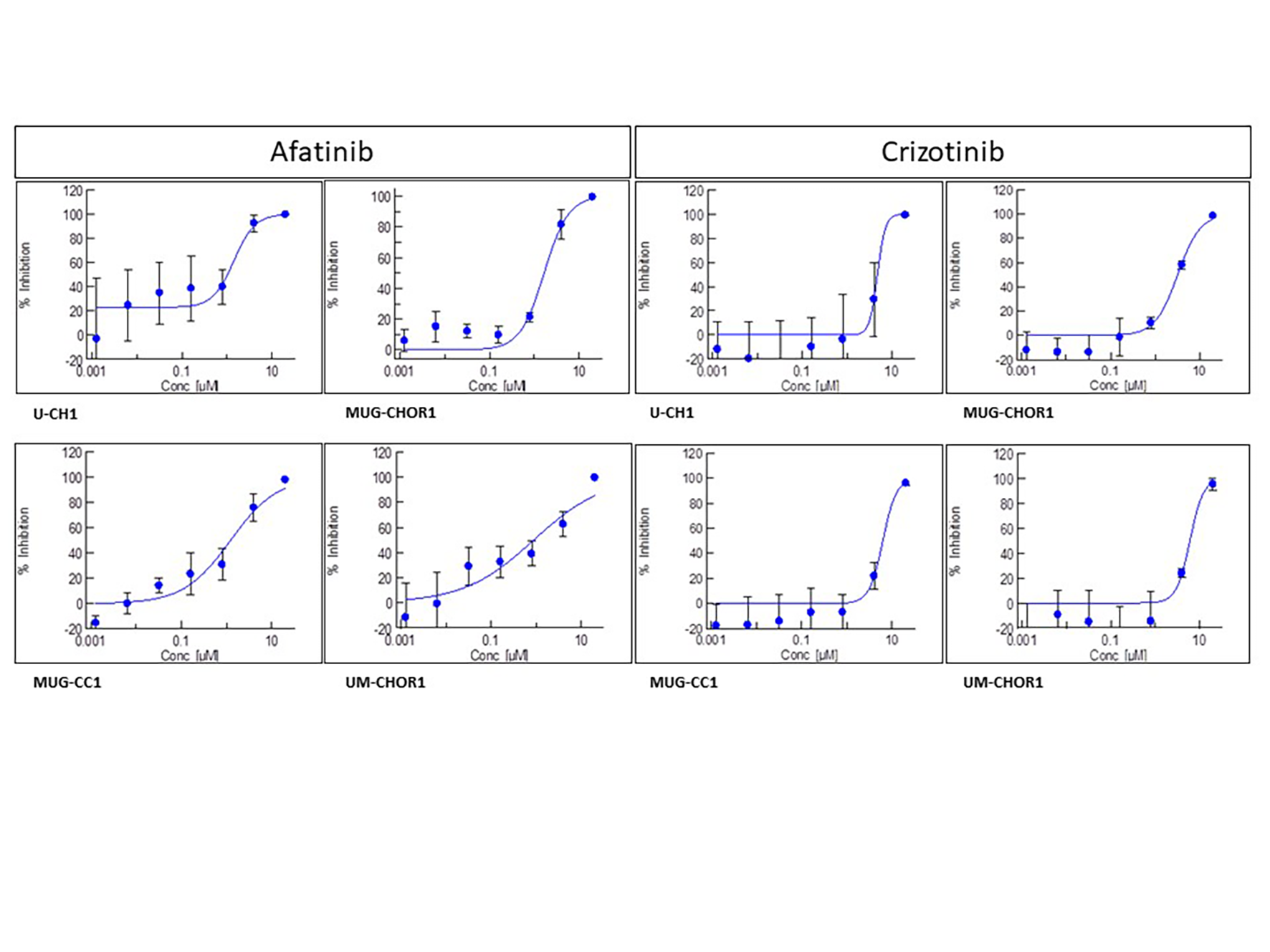

Supplement: Supplementary file 1 — The EGFRi afatinib indicates activity in all four chordoma cell lines utilised in this study (UM-Chor1, MUG-Chor1, U-CH1, and MUG-CC1). EC50 values vary between cell lines but are all within the nanomolar or low micromolar range. The ALK/MET-inhibitor crizotinib is less potent as a single agent than afatinib. The EC50s of crizotinib are within a high-micromolar range in all four cell lines. (PNG 487 kb) [file 13402_2021_632_Fig7_ESM.png]

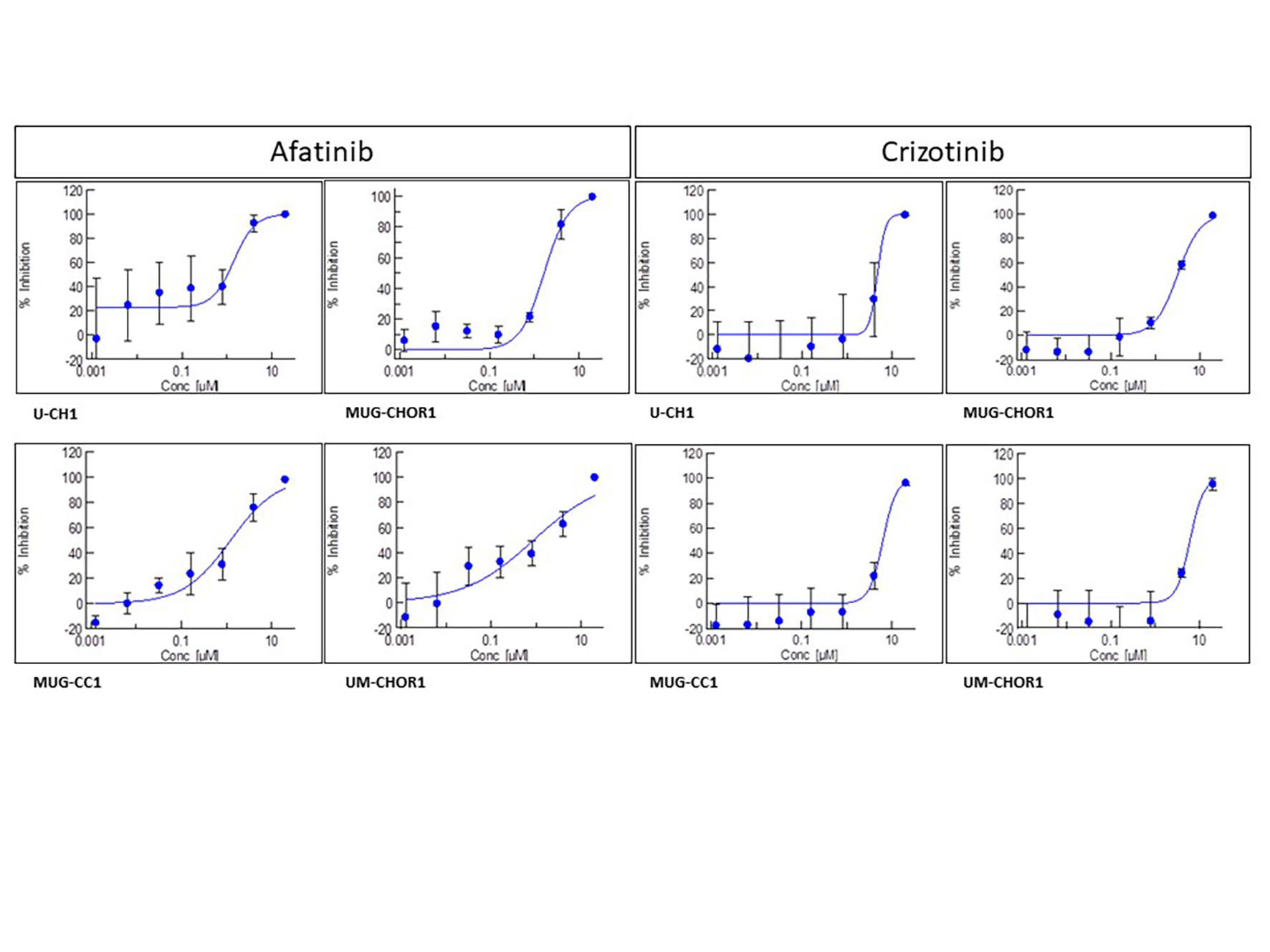

Supplement: Supplementary file 2 — High resolution image (TIF 674 kb) [file 13402_2021_632_MOESM1_ESM.tif]
