# Supplementary material for: Drug combination screening as a translational approach toward an improved drug therapy for chordoma
Source: Cell Oncol (Dordr). 2021 Sep 22;44(6):1231–42. doi: 10.1007/s13402-021-00632-x (PMC8648636; doi:10.1007/s13402-021-00632-x)
Supplement: Supplementary file 3 — STR authentication profiles of chordoma cell lines utilised in this study. (DOCX 15 kb) [file 13402_2021_632_MOESM2_ESM.docx]

**Suppl. Table 1. STR profiles of chordoma cell lines.**

| **STR-Analysis** | **U-CH1** | **UM-Chor1** | **MUG-Chor1** | **MUG-CC1** |
| --- | --- | --- | --- | --- |
| **Marker** |  |  |  |  |
| **D3S1358** | 15 | 18 | 14, 17 | 16 |
| **TH01** | 7 | 7, 9.3 | 9.3 | 9.3 |
| **D21S11** | 28, 29 | 27, 31 | 29, 33.2 | 28 |
| **D18S51** | 15 | 14 | 17, 23 | 12 |
| **Penta E** | 7, 10 | 7 | 5, 12 | 10 |
| **D5S818** | 11, 12 | 9, 13 | 11, 12 | 11, 12 |
| **D13S137** | 11, 13 | 12 | 11 | 12, 13 |
| **D7S820** | 9, 12 | 11 | 8, 11 | 8, 11 |
| **D16S539** | 12, 13 | 12 | 11, 14 | 9, 14 |
| **CSF1PO** | 10, 11 | 11 | 11 | 11 |
| **Penta D** | 11 | 8, 9 | 13 | 13 |
| **AMEL** | X, Y | X, Y | X | X |
| **vWA** | 17 | 15 | 15 | 16, 18 |
| **D8S1179** | 10, 15 | 12, 13 | 11, 12 | 13, 15 |
| **TPOX** | 8, 11 | 8, 9 | 8 | 8, 11 |
| **FGA** | 20, 21 | 23 | 21, 26 | 23 |
