# Supplementary material for: Drug combination screening as a translational approach toward an improved drug therapy for chordoma
Source: Cell Oncol (Dordr). 2021 Sep 22;44(6):1231–42. doi: 10.1007/s13402-021-00632-x (PMC8648636; doi:10.1007/s13402-021-00632-x)
Supplement: Supplementary file 4 — Panel of 133 U.S. Food and Drug Administration (FDA)-approved anticancer drugs that was kindly provided to us by the National Institute of Health (NIH) Cancer Institute, Developmental Therapeutics Programme (DTP), Bethesda, Maryland, USA. NSC: NSC number (Cancer Chemotherapy National Service Centre number); identifying number assigned by DTP. CAS: CAS Registry Number. MW: molecular weight. (DOCX 40 kb) [file 13402_2021_632_MOESM3_ESM.docx]

**Suppl. Table 2. Summary of compounds included in the single point screen (n=133).**

|  | **Plate ID** | **Well ID** | **Cpd. Nr.** | **NSC*** | **CAS**** | **Drug Name (USAN)** | **Molecular Weight** | **Plate Nr.** |
| --- | --- | --- | --- | --- | --- | --- | --- | --- |
| **Nr.** |  |  |  |  |  |  |  |  |
| 1 | 4858 | A02 | 2 | 1390 | 315-30-0 | Allopurinol | 136,11 | Plate 1 |
| 2 | 4858 | A03 | 3 | 45388 | 4342-03-4 | Dacarbazine | 182,18 | Plate 1 |
| 3 | 4858 | A04 | 4 | 102816 | 320-67-2 | Azacitidine | 244,21 | Plate 1 |
| 4 | 4858 | A05 | 5 | 77213 | 366-70-1 | Procarbazine hydrochloride | 257,76 | Plate 1 |
| 5 | 4858 | A06 | 6 | 613327 | 122111-03-9 | Gemcitabine hydrochloride | 299,65 | Plate 1 |
| 6 | 4858 | A07 | 7 | 3088 | 305-03-3 | Chlorambucil | 304,22 | Plate 1 |
| 7 | 4858 | A08 | 8 | 71423 | 595-33-5 | Megestrol acetate | 384,51 | Plate 1 |
| 8 | 4858 | A09 | 9 | 754355 | 1038915-60-4 | Niraparib hydrochloride | 356,85 | Plate 1 |
| 9 | 4858 | A10 | 10 | 755980 | 417716-92-8 | Lenvatinib | 426,86 | Plate 1 |
| 10 | 4858 | A11 | 11 | 609699 | 119413-54-6 | Topotecan hydrochloride | 457,91 | Plate 1 |
| 11 | 4858 | B02 | 14 | 19893 | 51-21-8 | Fluorouracil | 130,08 | Plate 1 |
| 12 | 4858 | B03 | 15 | 92859 | 1327-53-3 | Arsenic trioxide | 197,84 | Plate 1 |
| 13 | 4858 | B04 | 16 | 127716 | 2353-33-5 | Decitabine | 228,21 | Plate 1 |
| 14 | 4858 | B05 | 17 | 85998 | 18883-66-4 | Streptozocin | 265,22 | Plate 1 |
| 15 | 4858 | B06 | 18 | 701852 | 149647-78-9 | Vorinostat | 264,32 | Plate 1 |
| 16 | 4858 | B07 | 19 | 8806 | 3223-07-2 | Melphalan hydrochloride | 341,66 | Plate 1 |
| 17 | 4858 | B08 | 20 | 138783 | 3543-75-7 | Bendamustine hydrochloride | 394,73 | Plate 1 |
| 18 | 4858 | B09 | 21 | 756655 | 179324-69-7 | Bortezomib | 384,24 | Plate 1 |
| 19 | 4858 | B10 | 22 | 755986 | 879085-55-9 | Vismodegib | 421,30 | Plate 1 |
| 20 | 4858 | B11 | 23 | 732517 | 863127-77-9 | Dasatinib | 488,01 | Plate 1 |
| 21 | 4858 | C02 | 26 | 32065 | 127-07-1 | Hydroxyurea | 76,05 | Plate 1 |
| 22 | 4858 | C03 | 27 | 362856 | 85622-93-1 | Temozolomide | 194,15 | Plate 1 |
| 23 | 4858 | C04 | 28 | 409962 | 154-93-8 | Carmustine | 214,05 | Plate 1 |
| 24 | 4858 | C05 | 29 | 105014 | 4291-63-8 | Cladribine | 285,69 | Plate 1 |
| 25 | 4858 | C06 | 30 | 713563 | 107868-30-4 | Exemestane | 296,40 | Plate 1 |
| 26 | 4858 | C07 | 31 | 26980 | 50-07-7 | Mitomycin | 334,33 | Plate 1 |
| 27 | 4858 | C08 | 32 | 241240 | 41575-94-4 | Carboplatin | 371,25 | Plate 1 |
| 28 | 4858 | C09 | 33 | 757441 | 319460-85-0 | Axitinib | 386,47 | Plate 1 |
| 29 | 4858 | C10 | 34 | 756644 | 459868-92-9 | Rucaparib phosphate | 421,36 | Plate 1 |
| 30 | 4858 | C11 | 35 | 743414 | 152459-95-5 | Imatinib | 493,61 | Plate 1 |
| 31 | 4858 | D02 | 38 | 752 | 154-42-7 | Thioguanine | 167,19 | Plate 1 |
| 32 | 4858 | D03 | 39 | 750 | 55-98-1 | Busulfan | 246,30 | Plate 1 |
| 33 | 4858 | D04 | 40 | 26271 | 6055-19-2 | Cyclophosphamide | 261,09 | Plate 1 |
| 34 | 4858 | D05 | 41 | 109724 | 3778-73-2 | Ifosfamide | 261,09 | Plate 1 |
| 35 | 4858 | D06 | 42 | 719344 | 120511-73-1 | Anastrozole | 293,37 | Plate 1 |
| 36 | 4858 | D07 | 43 | 38721 | 53-19-0 | Mitotane | 320,04 | Plate 1 |
| 37 | 4858 | D08 | 44 | 266046 | 61825-94-3 | Oxaliplatin | 397,29 | Plate 1 |
| 38 | 4858 | D09 | 45 | 788948 | 4105-38-8 | Uridine triacetate | 370,32 | Plate 1 |
| 39 | 4858 | D10 | 46 | 756645 | 877399-52-5 | Crizotinib | 450,34 | Plate 1 |
| 40 | 4858 | D11 | 47 | 747971 | 284461-73-0 | Sorafenib | 464,82 | Plate 1 |
| 41 | 4858 | E02 | 50 | 755 | 50-44-2 | Mercaptopurine | 152,18 | Plate 1 |
| 42 | 4858 | E03 | 51 | 13875 | 645-05-6 | Altretamine | 210,28 | Plate 1 |
| 43 | 4858 | E04 | 52 | 34462 | 66-75-1 | Uracil mustard | 252,10 | Plate 1 |
| 44 | 4858 | E05 | 53 | 119875 | 15663-27-1 | Cisplatin | 300,06 | Plate 1 |
| 45 | 4858 | E06 | 54 | 719345 | 112809-51-5 | Letrozole | 285,30 | Plate 1 |
| 46 | 4858 | E07 | 55 | 606869 | 123318-82-1 | Clofarabine | 303,68 | Plate 1 |
| 47 | 4858 | E08 | 56 | 312887 | 75607-67-9 | Fludarabine phosphate | 365,21 | Plate 1 |
| 48 | 4858 | E09 | 57 | 279836 | 65271-80-9 | Mitoxantrone | 444,49 | Plate 1 |
| 49 | 4858 | E10 | 58 | 759224 | 870281-82-6 | Idelalisib | 415,42 | Plate 1 |
| 50 | 4858 | E11 | 59 | 747974 | 84449-90-1 | Raloxifene | 473,59 | Plate 1 |
| 51 | 4858 | F02 | 62 | 762 | 55-86-7 | Mechlorethamine hydrochloride | 192,52 | Plate 1 |
| 52 | 4858 | F03 | 63 | 27640 | 50-91-9 | Floxuridine | 246,19 | Plate 1 |
| 53 | 4858 | F04 | 64 | 63878 | 69-74-9 | Cytarabine hydrochloride | 279,70 | Plate 1 |
| 54 | 4858 | F05 | 65 | 122758 | 302-79-4 | Tretinoin | 300,44 | Plate 1 |
| 55 | 4858 | F06 | 66 | 747972 | 191732-72-6 | Lenalidomide | 259,26 | Plate 1 |
| 56 | 4858 | F07 | 67 | 758774 | 414864-00-9 | Belinostat | 318,35 | Plate 1 |
| 57 | 4858 | F08 | 68 | 712807 | 154361-50-9 | Capecitabine | 359,35 | Plate 1 |
| 58 | 4858 | F09 | 69 | 715055 | 184475-35-2 | Gefitinib | 446,90 | Plate 1 |
| 59 | 4858 | F10 | 70 | 761910 | 936563-96-1 | Ibrutinib | 440,50 | Plate 1 |
| 60 | 4858 | F11 | 71 | 750691 | 439081-18-2 | Afatinib | 485,94 | Plate 1 |
| 61 | 4858 | G02 | 74 | 6396 | 52-24-4 | Thiotepa | 189,22 | Plate 1 |
| 62 | 4858 | G03 | 75 | 45923 | 298-81-7 | Methoxsalen | 216,19 | Plate 1 |
| 63 | 4858 | G04 | 76 | 66847 | 50-35-1 | Thalidomide | 258,23 | Plate 1 |
| 64 | 4858 | G05 | 77 | 169780 | 24584-09-6 | Dexrazoxane | 268,27 | Plate 1 |
| 65 | 4858 | G06 | 78 | 755985 | 121032-29-9 | Nelarabine | 297,27 | Plate 1 |
| 66 | 4858 | G07 | 79 | 761190 | 404950-80-7 | Panobinostat | 349,43 | Plate 1 |
| 67 | 4858 | G08 | 80 | 719627 | 169590-42-5 | Celecoxib | 381,37 | Plate 1 |
| 68 | 4858 | G09 | 81 | 718781 | 183319-69-9 | Erlotinib hydrochloride | 429,90 | Plate 1 |
| 69 | 4858 | G10 | 82 | 778909 | 1211441-98-3 | Ribociclib | 434,54 | Plate 1 |
| 70 | 4858 | G11 | 83 | 754230 | 146464-95-1 | Pralatrexate | 477,48 | Plate 1 |
| 71 | 4858 | H02 | 86 | 18509 | 5451-09-2 | Aminolevulinic acid hydrochloride | 167,59 | Plate 1 |
| 72 | 4858 | H03 | 87 | 79037 | 13010-47-4 | Lomustine | 233,70 | Plate 1 |
| 73 | 4858 | H04 | 88 | 75520 | 70-00-8 | Trifluridine | 296,20 | Plate 1 |
| 74 | 4858 | H05 | 89 | 218321 | 53910-25-1 | Pentostatin | 268,27 | Plate 1 |
| 75 | 4858 | H06 | 90 | 775351 | 19171-19-8 | Pomalidomide | 273,25 | Plate 1 |
| 76 | 4858 | H07 | 91 | 25154 | 54-91-1 | Pipobroman | 356,06 | Plate 1 |
| 77 | 4858 | H08 | 92 | 750690 | 557795-19-4 | Sunitinib | 398,47 | Plate 1 |
| 78 | 4858 | H09 | 93 | 753686 | 763113-22-0 | Olaparib | 434,46 | Plate 1 |
| 79 | 4858 | H10 | 94 | 740 | 59-05-2 | Methotrexate | 454,44 | Plate 1 |
| 80 | 4858 | H11 | 95 | 755384 | 357166-30-4 | Pemetrexed, Disodium salt, Heptahydrate | 471,38 | Plate 1 |
| 81 | 4859 | A02 | 2 | 755605 | 915087-33-1 | Enzalutamide | 464,42 | Plate 2 |
| 82 | 4859 | A03 | 3 | 754143 | 128517-07-7 | Romidepsin | 540,69 | Plate 2 |
| 83 | 4859 | A04 | 4 | 123127 | 25316-40-9 | Doxorubicin hydrochloride | 579,99 | Plate 2 |
| 84 | 4859 | A05 | 5 | 24559 | 18378-89-7 | Plicamycin | 1085,16 | Plate 2 |
| 85 | 4859 | A06 | 6 | 608210 | 125317-39-7 | Vinorelbine tartrate | 1079,00 | Plate 2 |
| 86 | 4859 | A07 | 7 | 761432 | 183133-96-2 | Cabazitaxel | 835,94 | Plate 2 |
| 87 | 4859 | A08 | 8 | 749226 | 154229-19-3 | Abiraterone | 349,51 | Plate 2 |
| 88 | 4859 | B02 | 14 | 760766 | 443913-73-3 | Vandetanib | 475,36 | Plate 2 |
| 89 | 4859 | B03 | 15 | 758253 | 26833-87-4 | Omacetaxine mepesuccinate | 545,63 | Plate 2 |
| 90 | 4859 | B04 | 16 | 141540 | 33419-42-0 | Etoposide | 588,56 | Plate 2 |
| 91 | 4859 | B05 | 17 | 49842 | 143-67-9 | Vinblastine sulfate | 909,06 | Plate 2 |
| 92 | 4859 | B06 | 18 | 616348 | 100286-90-6 | Irinotecan hydrochloride | 623,15 | Plate 2 |
| 93 | 4859 | B07 | 19 | 764134 | 1195768-06-9 | Dabrafenib mesylate | 615,65 | Plate 2 |
| 94 | 4859 | B08 | 20 | 296961 | 20537-88-6 | Amifostine | 214,22 | Plate 2 |
| 95 | 4859 | C02 | 26 | 761385 | 956697-53-3 | Erismodegib | 485,49 | Plate 2 |
| 96 | 4859 | C03 | 27 | 758254 | 1201902-80-8 | Ixazomib citrate | 517,13 | Plate 2 |
| 97 | 4859 | C04 | 28 | 180973 | 54965-24-1 | Tamoxifen citrate | 563,65 | Plate 2 |
| 98 | 4859 | C05 | 29 | 67574 | 2068-78-2 | Vincristine sulfate | 923,04 | Plate 2 |
| 99 | 4859 | C06 | 30 | 628503 | 114977-28-5 | Docetaxel | 807,89 | Plate 2 |
| 100 | 4859 | C07 | 31 | 766270 | 1257044-40-8 | Venetoclax | 868,45 | Plate 2 |
| 101 | 4859 | C08 | 32 | 737754 | 635702-64-6 | Pazopanib hydrochloride | 473,98 | Plate 2 |
| 102 | 4859 | D02 | 38 | 761431 | 1029872-54-5 | Vemurafenib | 489,91 | Plate 2 |
| 103 | 4859 | D03 | 39 | 758487 | 943319-70-8 | Ponatinib | 532,55 | Plate 2 |
| 104 | 4859 | D04 | 40 | 256942 | 56390-09-1 | Epirubicin hydrochloride | 579,99 | Plate 2 |
| 105 | 4859 | D05 | 41 | 122819 | 29767-20-2 | Teniposide | 656,66 | Plate 2 |
| 106 | 4859 | D06 | 42 | 683864 | 162635-04-3 | Temsirolimus | 1030,29 | Plate 2 |
| 107 | 4859 | D07 | 43 | 369100 | 99011-02-6 | Imiquimod | 240,31 | Plate 2 |
| 108 | 4859 | D08 | 44 | 747599 | 641571-10-0 | Nilotinib | 529,51 | Plate 2 |
| 109 | 4859 | E02 | 50 | 763932 | 755037-03-7 | Regorafenib | 482,80 | Plate 2 |
| 110 | 4859 | E03 | 51 | 761068 | 849217-68-1 | Cabozantinib | 501,51 | Plate 2 |
| 111 | 4859 | E04 | 52 | 702294 | 52205-73-9 | Estramustine phosphate sodium | 564,35 | Plate 2 |
| 112 | 4859 | E05 | 53 | 125066 | 9041-93-4 | Bleomycin sulfate | 1512,61 | Plate 2 |
| 113 | 4859 | E06 | 54 | 719276 | 129453-61-8 | Fulvestrant | 606,75 | Plate 2 |
| 114 | 4859 | E07 | 55 | 758247 | 571190-30-2 | Palbociclib | 447,54 | Plate 2 |
| 115 | 4859 | E08 | 56 | 764040 | 1256580-46-7 | Alectinib | 482,62 | Plate 2 |
| 116 | 4859 | F02 | 62 | 779217 | 1421373-65-0 | Osimertinib | 499,61 | Plate 2 |
| 117 | 4859 | F03 | 63 | 765694 | 380843-75-4 | Bosutinib | 530,45 | Plate 2 |
| 118 | 4859 | F04 | 64 | 745750 | 231277-92-2 | Lapatinib | 581,06 | Plate 2 |
| 119 | 4859 | F05 | 65 | 125973 | 33069-62-4 | Paclitaxel | 853,92 | Plate 2 |
| 120 | 4859 | F06 | 66 | 733504 | 159351-69-6 | Everolimus | 958,24 | Plate 2 |
| 121 | 4859 | F07 | 67 | 761388 | 110078-46-1 | Plerixafor | 502,79 | Plate 2 |
| 122 | 4859 | G02 | 74 | 256439 | 57852-57-0 | Idarubicin hydrochloride | 533,96 | Plate 2 |
| 123 | 4859 | G03 | 75 | 768068 | 934660-93-2 | Cobimetinib | 531,32 | Plate 2 |
| 124 | 4859 | G04 | 76 | 776422 | 1032900-25-6 | Ceritinib | 558,14 | Plate 2 |
| 125 | 4859 | G05 | 77 | 226080 | 53123-88-9 | Sirolimus | 914,18 | Plate 2 |
| 126 | 4859 | G06 | 78 | 758246 | 871700-17-3 | Trametinib | 615,40 | Plate 2 |
| 127 | 4859 | G07 | 79 | 9706 | 51-18-3 | Triethylenemelamine | 204,23 | Plate 2 |
| 128 | 4859 | H02 | 86 | 747973 | 219989-84-1 | Ixabepilone | 506,70 | Plate 2 |
| 129 | 4859 | H03 | 87 | 82151 | 23541-50-6 | Daunorubicin hydrochloride | 563,98 | Plate 2 |
| 130 | 4859 | H04 | 88 | 3053 | 50-76-0 | Dactinomycin | 1255,43 | Plate 2 |
| 131 | 4859 | H05 | 89 | 246131 | 56124-62-0 | Valrubicin | 723,64 | Plate 2 |
| 132 | 4859 | H06 | 90 | 758252 | 868540-17-4 | Carfilzomib | 719,92 | Plate 2 |
| 133 | 4859 | H07 | 91 | 721517 | 118072-93-8 | Zoledronic acid | 272,09 | Plate 2 |

DTP: Developmental Therapeutics Program, National Institutes of Health, Bethesda, MD, USA

**NSC: NSC number (Cancer Chemotherapy National Service Center number); identifying number assigned by DTP

*CAS Registry Number: Chemical Abstracts Service
